# Central fragmentation of an orthokeratology lens: Improper lens care

**DOI:** 10.5935/0004-2749.2024-0128

**Published:** 2024-10-23

**Authors:** Weimin Zhang, Shujuan Wang, Yingcheng Lin, Min Zhou

**Affiliations:** 1 Department of Ophthalmology, Jingliang Eye Hospital, Guangxi Medical University, Nanning, Guangxi, 530000, P. R. China; 2 Guangxi University of Chinese Medicine, Nanning, Guangxi, 530200, P. R. China; 3 Department of Ophthalmology, Taoyuan Hospital of Traditional Chinese Medicine, Taoyuan, Hunan, 415700, P. R. China

**Figure:**
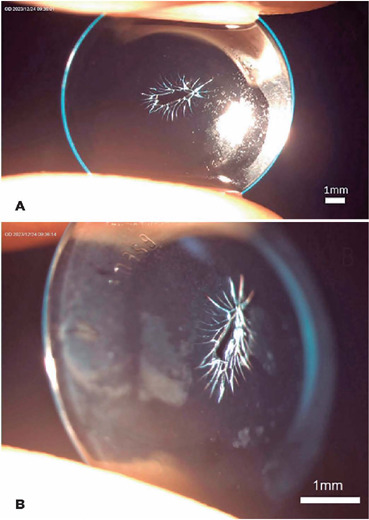

An 11-year-old girl presented to our clinic with a fractured lens in her right eye following two years of orthokeratology lens wear. The central area of the lens appeared feathery, indicating damage, while the surrounding area was unaffected. Fluorescence staining confirmed the corneal integrity, indicating that the damage was the result of the negative pressure generated when using a tool to remove the lens from the central region. This highlights the importance of proper care and handling during the fitting and maintenance of orthokeratology lenses^(1)^. Contact lens users are predisposed to complications such as bacterial colonization^(2)^, corneal abrasions^(3)^, and suboptimal outcomes. Therefore, proper lens maintenance and adherence to careful application and removal techniques are crucial. The lens should be removed from the periphery to avoid generating excessive negative pressure at the center, thereby preventing corneal adhesions and potential damage^(4)^.

The image shows the significant damage at the center of the orthokeratology lens, with a feathery appearance on the anterior (side A) and lateral (side B) views. The lens features a corneal refractive therapy (CRT) design^(5)^ that is characterized by a treatment curve radius of 8.2 mm, return zone depth of 600 µm, and landing zone angle of 32°.
